# Integrating HIV-Associated Neurocognitive Impairment Screening within Primary Healthcare Facilities: A Pilot Training Intervention

**DOI:** 10.1155/2022/4495586

**Published:** 2022-08-13

**Authors:** Adele Munsami, Goodman Sibeko, Hetta Gouse, Sam Nightingale, John A. Joska

**Affiliations:** HIV Mental Health Research Unit, Division of Neuropsychiatry, Department of Psychiatry and Mental Health, University of Cape Town, Cape Town, South Africa

## Abstract

HIV-associated neurocognitive impairment (H-NCI) remains a common comorbidity, which may affect several key health outcomes among people with HIV. However, there are shortages of appropriately skilled healthcare workers able to identify and manage H-NCI in low- and middle-income countries. We conducted an exploratory, quasi-experimental, pre- and post-cohort training intervention in KwaZulu-Natal, South Africa. Thirty-four healthcare workers (two general medical doctors, twenty-two nurses, and ten adherence counsellors) from six facilities and a mobile clinic unit attended two, two-hour face-to-face, training sessions. The training included knowledge and skill transfer components. Pre- and post-knowledge questionaries demonstrated an improvement among 82% (*n* = 28) of the attendees from all three cadres. Knowledge was retained by 88% (*n* = 30) of the attendees after eight weeks. The H-NCI screening tools were administered with 78% accuracy. After eight weeks, two general medical doctors and eight senior nurses were able to accurately administer the tool. The Primary Healthcare H-NCI training was successful in improving knowledge among primary healthcare workers; however, several healthcare workers experienced challenges with administering such tools.

## 1. Introduction

HIV is now recognized as a manageable, multisystem, chronic illness, following the widespread availability of virologically suppressive antiretroviral therapy (ART) [1]. More than two-thirds of the global population of people with HIV (PWH) are found in sub-Saharan Africa [2]. South Africa is the country most severely affected by the epidemic, accounting for approximately 8.2 million PWH. South Africa is also home to the largest ART program worldwide, with 72% of PWH in the country currently accessing treatment [3, 4]. The overall HIV prevalence rate is approximately 13,7% among the South African population [3].

Over the past decade, there have been significant declines in several AIDS-defining illnesses, including severe HIV-associated neurocognitive impairment (H-NCI), such as HIV-associated dementia (HIV-D) [1]. However, while the incidence of HIV-D has fallen dramatically, the lifetime prevalence may remain stable or even increase as PWH on effective ART experience longer life expectancies [5].

PWH with symptoms of H-NCI experience difficulties with attention, memory, learning, problem solving, decision making, and activities required for everyday functioning, including ART adherence [6]. Healthcare providers in clinical settings have been noted to have limited knowledge of H-NCI, and screening practices remain uncommon in low- and middle-income countries [7–9]. Reasons for low H-NCI screening may be underdetection due to low incidence and prevalence rates in the context of effective ART, poor knowledge, and screening skills among healthcare workers in this setting, limited skilled personnel, treatment alternatives, and/or medical costs associated with neurocognitive care.

Low- and middle-income countries experience severe shortages in trained healthcare providers, especially mental and neurological healthcare workers [10]. The medical, surgical, and neurosurgical specialties have adopted task-sharing to address human resource shortages in this setting [11]. This approach involves the redistribution of duties through delegation [12]. Several healthcare workers from various tiers form a collaborative team of specialists and less-qualified cadres, relying on iterative communication, as well as ongoing training to preserve high-quality outcomes [12].

It is unclear whether targeted H-NCI screening for neurocognitive and functional symptoms can be effectively integrated into a primary healthcare clinic through task-sharing from specialists to primary healthcare workers [10, 13]. A systematic review conducted by Liu et al. [14] reported several studies exploring task-sharing through the delegation of mental health services from specialists to general medical doctors, nurses, and community healthcare workers. However, few studies included H-NCI in the training interventions [14]. Although one H-NCI training intervention found improved confidence in identifying H-NCI symptoms among clinical officers in Kenya, this training did not include general medical doctors, nurses, and adherence counsellors [10]. Task-sharing, which is commonly evaluated through training, practice, and maintenance, has not been explored among general medical doctors, nurses, and/or adherence counsellors administering H-NCI screening in this setting [12].

Our study aimed to provide preliminary evidence needed to inform the design of an H-NCI training intervention targeting primary healthcare workers in low- and middle-income countries. The aim of this intervention is determine whether task-sharing of H-NCI screening from specialists to primary healthcare workers would be feasible in this setting. Our primary objective was to explore the viability of H-NCI screening by primary healthcare workers. We examined the initial uptake of knowledge and skills among general medical doctors, nurses, and adherence counsellors following the training intervention. Our secondary objective was to examine the appropriateness of an in-field, H-NCI training intervention among primary healthcare workers at this level of care.

## 2. Methods

### 2.1. Study Design

An exploratory, quasi-experimental, pre- and post-cohort intervention study was conducted among primary healthcare workers. These healthcare workers provide the majority of HIV services in KwaZulu-Natal, South Africa. We developed a four-hour training programme that was spread over two weekly, two-hour sessions. The programme design comprised didactic lectures for knowledge and H-NCI skill transfer delivered over a short period. This was with a view toward making the training cost-effective and easily replicated by future interventions in low- and middle-income countries. The on-site design also ensured that the healthcare workers remained in the facility and were available to deal with emergencies, thus ensuring minimal impact on patient services in already burdened facilities. The development process of the pilot H-NCI training is described.

The training sessions took place at primary healthcare facilities based on the principles of academic detailing. Academic detailing involves peer-to-peer outreach processes involving visits to the work setting of a target audience from a trained professional. The approach allows the trainer to demonstrate and discuss topics including new skill sets to improve patient services [15]. The main aim of academic detailing is to increase the uptake of a new intervention, such as the use of a standardized assessment tool in community practice, which is otherwise uncommon [15, 16]. The iterative processes of academic detailing involving peer-to-peer skill transfer, demonstration, and feedback were appropriate for our pilot training.

### 2.2. Ethical Considerations

The University of Cape Town Faculty of Health Sciences Human Research Ethics Committee, the City of Cape Town Department of Health, and the KZN Department of Health in the Ugu District provided approval for this study. The training was approved by the HIV and Sexually Transmitted Infections (HAST) district manager, district mental health manager, the operational manager, and the chief executive officer at each facility. All participants provided informed consent prior to participation.

### 2.3. Population and Sample

We applied the general rule which suggests a minimum of 30 subjects or greater for pilot studies [17]. This sample size was sufficient to evaluate procedures and processes which will inform future training interventions. To recruit participants for the Primary Healthcare H-NCI training, we approached medical managers from the HAST units at primary healthcare facilities in the Ugu district under the KwaZulu-Natal Department of Health. General medical doctors, nurses, and adherence counsellors working in these units were invited to participate in the training. The healthcare workers were informed by the medical manager that participation was not mandatory. Between June and July 2021, 34 primary healthcare workers (2 doctors, 22 nurses, and 10 adherence counsellors) from five primary healthcare facilities and a mobile clinic unit attended both sessions of the pilot training.

### 2.4. Curriculum Development

The Primary Healthcare H-NCI training curriculum was developed by the first author (AM) and senior researchers from the University of Cape Town (JJ, HG, SN, and GS).  Table S1(supplementary material) provides an outline of the training curriculum. We used a participatory curriculum design approach to evaluate the training curriculum [18]. This approach relies on the involvement of stakeholders and end users in the design process, as well as facilitating buy-in from key personnel [18, 19]. The Primary Healthcare H-NCI training curriculum was evaluated by the HAST district manager who provides continued education to healthcare workers, the district mental health manager, and trainers from the various districts. These key stakeholders provided feedback and input to ensure that the training would be appropriate.

The first author developed a workbook to facilitate learning during the training, in consultation with the other authors (GS, HG, SN, and JJ). The workbook included the training content. Additional reading material providing more information that could not be covered, such as a detailed background of H-NCI was also provided in this workbook. The content of the workbook was summarised in point form and was presented to the training recipients by the first author using a power point presentation. This was guided by a trainer's manual developed by the first author with input from the co-authors. The trainer's manual included session directions, a speech guide for the trainer to follow and a list of required materials for each session. Visual aids in the form of video demonstrations of the screening tools were used to supplement the information provided in the workbook.

We used the International HIV Dementia Scale (IHDS) and the Cognitive Assessment Tool-rapid version (CAT-rapid) for this training. These tools have been validated for use in South Africa [20, 21]. The IHDS tests three neurocognitive domains, including motor speed (timed finger tapping), psychomotor speed and processing (timed alternating hand sequence test), as well as short-term memory (recollection of four words in two minutes) [22]. The CAT-rapid includes four symptom questions, short-term memory (registration of four words), executive functioning (a mini-trail-making test), psychomotor speed and processing (timed alternating hand sequence test) and verbal learning and memory (word recall) assessments [20]. A detailed description of the administration of the IHDS and the CAT-rapid is described by Sacktor et al. [22] and Witten [23].

### 2.5. Data Collection

#### 2.5.1. Delivery of the Training

The first author (AM) delivered the training. The first author has experience in HIV/TB training facilitation among general medical doctors, nurses, and adherence counsellors providing healthcare services at various levels in South Africa. The first author received training on neuropsychological screening tools whilst working on a research study conducted at the University of Cape Town, under the supervision of HG, who is a registered neuropsychologist. The Primary Healthcare H-NCI training sessions comprised didactic lectures, case vignettes, interactive discussions, and roleplaying. The first session focused on the theoretical components of H-NCI. The second session involved practical training on H-NCI screening tool administration.

#### 2.5.2. Participant Evaluation and Measures

The knowledge and skill assessments were designed using Miller's pyramid for assessing clinical competence [24, 25]. This framework has been used to assess clinical competence beyond test-taking or memorising of information [24]. The model emphasises the importance of acquiring knowledge to perform a task in practice [25]. The lowest level in the model is knowledge (knows), followed by competence (knows how), performance (shows how), and action (does) [24, 25]. We examined participants' attitudes and views of the H-NCI training in the post-training evaluation.

#### 2.5.3. Knowledge and Attitudes

Multiple-choice questionnaires were used to evaluate knowledge, as well as participants' attitude and comfort with H-NCI screening tools before and after the training. Pre- and post-testing is the most common technique adopted in academic detailing [15]. The current questions were adapted from a tool developed by Johnston et al. [26]. The tool consisted of 43 items, measuring nonskill attributes including knowledge, attitude, and behaviour among undergraduate medical students. A 20-item pre-test questionnaire was used to assess knowledge, attitudes, and views in the Primary Healthcare H-NCI training (Supplementary table S2). This tool was appropriate for our study as it sought to evaluate the teaching and learning of the medical curriculum.

#### 2.5.4. Skills

Direct observations of procedural skills (DOPS) were used to assess a healthcare worker's ability to administer the H-NCI screening tool. DOPS have been described by Wass et al. [27] as an appropriate assessment of clinical competence. Each trainee was assigned a one-on-one time slot to demonstrate the H-NCI screening tool on a standardized patient as part of a simulation. The simulations were assessed using a checklist detailing the steps required to accurately administer the selected screening tools and interpret the results. The participants' performances were scored by a counselling psychologist with previous experience in neuropsychological testing. Tasks that were accurately demonstrated were given a score of two, tasks that were listed on the checklist but were performed inaccurately were given a score of one and tasks that were incorrect or excluded were given a score of zero. Healthcare workers who did not accurately demonstrate the use of the screening tool were given immediate feedback by the trainer once the simulation was completed.

#### 2.5.5. Evaluation Procedures

**Figure 1:**
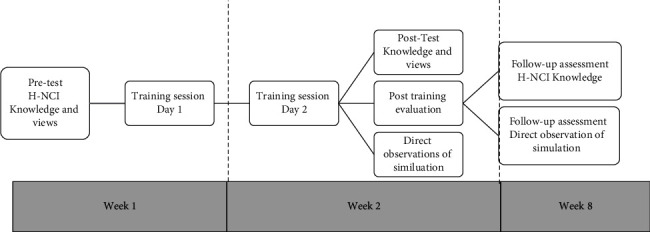
Flow diagram of HIV-associated NCI training and evaluations.

Data collected at baseline included participant demographics and pre-test questions. Knowledge of H-NCI and screening skills were assessed immediately after the training and after eight weeks. Training evaluation data were collected at the end of the second session. This is illustrated in the flow diagram (Figure 1). All data were collected anonymously using a unique participant identifier. Documentation containing participant names and their unique identifiers was kept confidential by using a hardcopy linking log that was stored in a separate location to the data.

### 2.6. Analysis

IBM Statistical Package for the Social Sciences (SPSS) 27 was used to analyse the data (IBM-Corp., 2017). We compared H-NCI knowledge among general medical doctors, nurses, and adherence counsellors before the training, immediately after the training, and eight weeks later. The data were analysed using frequency analysis and Fisher's exact *t*-test and are reported as the mean and standard deviation. We report healthcare workers' ability to administer H-NCI screening tools using chi-square analyses. We compared performance between the three cadres to establish which cadres would be appropriate to administer the H-NCI screening tools. We examined whether these skills were retained eight weeks after the training by comparing the three cadres.

## 3. Results

After describing participant characteristics, we present the results from the pre- and post-tests, DOPS, and follow-up assessments.

### 3.1. Participants

Of the 42 healthcare workers who initially enrolled to participate in the training, thirty-four participated in two training sessions (21 from facility one and 13 from facility two). Eight healthcare workers were excluded from the final analysis, including six who were required to address patients during the second session and two who were unavailable as they were scheduled to work the night shift on the day of the training.

Study participants consisted of two general medical doctors (6%), 22 nurses (65%), and ten adherence counsellors (29%) recruited from six primary healthcare facilities and a mobile health clinic. Most healthcare workers were females (71%, *n* = 24). The mean age of participants was 39 years (*SD* = 9.5, range: 26–62) and the median number of years in service was 12 years (*SD* = 7.8, range: 2–40).

### 3.2. H-NCI Knowledge Pre- and Post-Training

**Figure 2:**
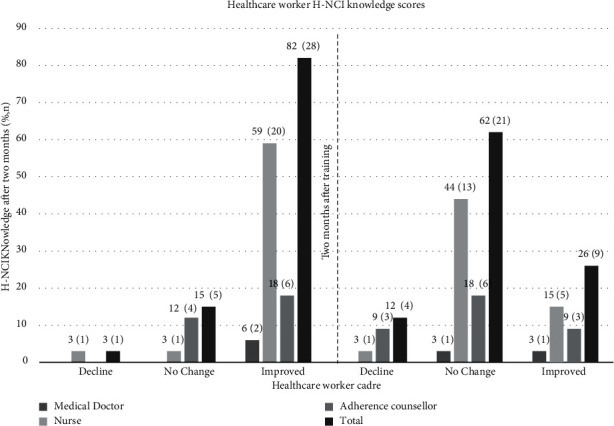
Healthcare workers' HIV-associated NCI knowledge scores before and after training.

To determine whether the pilot training intervention led to improved H-NCI knowledge among primary healthcare workers, contingency tables were used to compare pre-test questionnaire and post-test questionnaire scores for the total sample of 34 (Figure 2). The results showed that there was a significant increase in H-NCI knowledge overall. Prior to the training, the mean knowledge score was 61% (*SD* = 1.48). Healthcare workers demonstrated sufficient knowledge of H-NCI after the training (*M* = 83%, *SD* = 1.25). There were significant improvements in knowledge among 82% (*n* = 28). This was among all three cadres. Five (15%) healthcare workers (one nurse and four adherence counsellors) showed no change in knowledge, while one adherence counsellor (3%) scored lower as they intermittently dealt with patients during the training.

Figure 2 illustrates the change in knowledge scores between cadres eight weeks after the training intervention. Overall, 62% (*n* = 21) of healthcare workers scored the same before and after the training (one general medical doctor, 13 nurses, and six adherence counsellors). Nine (26%) healthcare workers (one general medical doctor, five nurses, and three adherence counsellors) demonstrated improved knowledge. There was a decline in knowledge among four (13%) healthcare workers (one nurse and three adherence counsellors).

**Figure 3:**
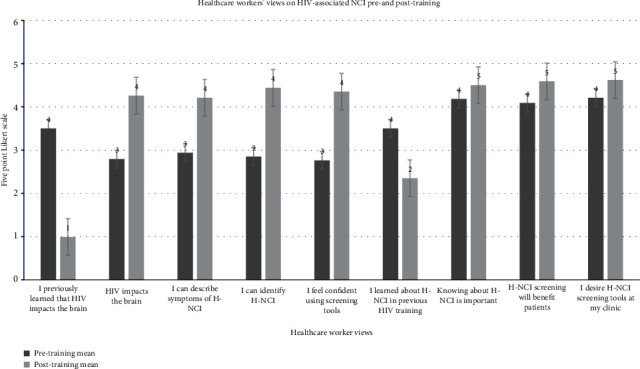
Healthcare worker views on HIV-associated NCI pre-training and post-training. *Note*: 5-point Likert scale. 0: strongly disagree, 1: disagree, 2: I do not know enough to answer, 3: agree, and 4: strongly agree

One-tail paired-sample *t*-tests were used to compare healthcare workers' views on their knowledge of H-NCI before and after the training. These results are illustrated in Figure 3. Those who had limited or no knowledge of the impact of HIV on the brain prior to training agreed that they had a significantly better understanding of the impact of HIV on the brain after the training. Prior to the training intervention, primary healthcare workers were unsure whether H-NCI was addressed in previous training. By the end of the H-NCI training, this was clarified and healthcare workers agreed that previous training did not address the impact of HIV on the brain or H-NCI.

### 3.3. Administering an H-NCI Screening Tool

#### 3.3.1. Overall Performance of Screening Tools Immediately after the Training

To understand whether primary healthcare workers would be able to accurately administer and interpret an H-NCI screening tool, we observed the demonstration of an H-NCI screening tool on a simulated patient. The mean performance scores are summarised in Table 1.

Overall, primary healthcare workers demonstrated the combined screening tool with 78% (*n* = 34) accuracy (*M* = 78.1, *SD* = 24.0). One general medical doctor and one nurse administered the combined screening tool with 100% accuracy (6%). Four (12%) healthcare workers (one general medical doctor and three nurses) demonstrated the screening tool with few errors, scoring between 96% and 99%. Overall, 41% (*n* = 14) of healthcare workers administered the screening tool with reasonable accuracy (range: 71%–95%), 21% (*n* = 7) administered the screening tool somewhat accurately (range: 40%–70%), and 6% (*n* = 2) administered the tool poorly (range: 0%–39%). The accuracy of the demonstrations varied among the three cadres. General medical doctors (91%, *n* = 2) and nurses (85%, *n* = 22) demonstrated the screening tools with greater accuracy compared to adherence counsellors (63%, *n* = 10).

#### 3.3.2. Healthcare Workers' Performance on the CAT-Rapid

Healthcare workers performed marginally better on the CAT-rapid screening tool (*M* = 79.0%, *SD* = 13.7) compared to the IHDS (*M* = 75.0%, *SD* = 11.4; see Table 1).

The CAT-rapid was administered with 100% accuracy by 15% (*n* = 5, one doctor and four nurses) of healthcare workers. Five (15%) healthcare workers (one general medical doctor and four nurses) demonstrated the CAT-rapid with few errors, scoring between 96% and 99%. Overall, 50% (*n* = 17) administered the CAT-rapid with reasonable accuracy (range: 71%–95%), 18% (*n* = 6) somewhat accurately (range: 40%–70%) and 12% (*n* = 4) administered the CAT-rapid poorly (range: 0%–39%). Overall, general medical doctors performed the CAT-rapid with 90% accuracy (*n* = 2), followed by nurses who demonstrated 84% accuracy (*n* = 22). The adherence counsellors (*n* = 10) demonstrated the CAT-rapid somewhat accurately (66%).

**Figure 4:**
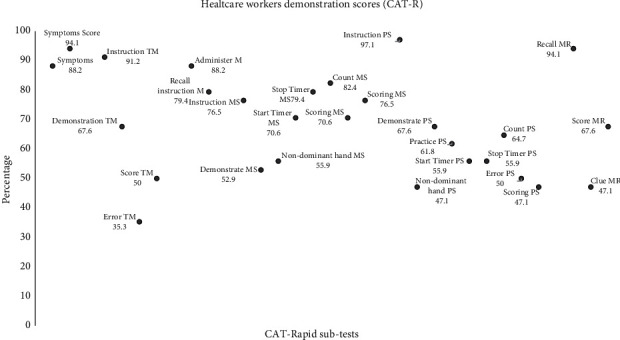
Healthcare workers' performance of the CAT-rapid subtests.

Our results show that primary healthcare workers were least successful with the following components of the CAT-rapid: demonstrating the timed finger tapping test, demonstrating the alternating hand sequence test, scoring the alternating hand sequence test, scoring the mini trail-making test, administering the memory recall clues, ensuring that the nondominant hand was used during the test and the use of the timer for the alternating hand sequence test (see Figure 4).

#### 3.3.3. Healthcare Workers' Performance on the IHDS

The IHDS was administered with 100% accuracy by 21% (*n* = 7) of primary healthcare workers (one general medical doctor and six nurses). Four (12%) healthcare workers (one general medical doctor and three nurses) demonstrated the IHDS with few errors, scoring between 96% and 99%. Overall, 38% of healthcare workers (*n* = 13) administered the IHDS with reasonable accuracy (range: 71%–95%), 29% (*n* = 10) somewhat accurately (range: 40%–70%) and 12% (*n* = 4) administered the IHDS poorly (range: 0%–39%). Between the cadres, the IHDS was demonstrated with greater accuracy by general medical doctors (90%) and nurses (82%). Adherence counsellors demonstrated the IHDS somewhat accurately (57%).

**Figure 5:**
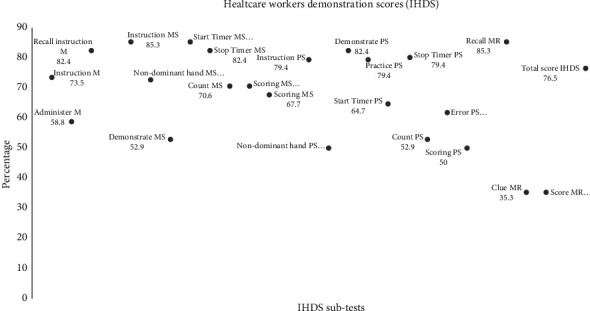
Healthcare workers' performance of the International HIV Dementia Scale subtests.

Primary healthcare workers were least successful with the following components of the IHDS (Figure 5): the administration of the memory recall clue, scoring the memory recall task, scoring the alternating hand sequence test and ensuring that the non-dominant hand was used during the test.

#### 3.3.4. Two-Month Posttraining

Primary healthcare workers were asked to demonstrate the screening tools eight weeks after the training. The results of the follow-up DOPS are summarised in Table 2.

Ten (29%) healthcare workers (one doctor and nine nurses) demonstrated the combined tool with 100% accuracy after eight weeks of daily practice. Overall, 41% (*n* = 14) of health care workers (one general medical doctor, ten nurses, and three adherence counsellors) improved on their demonstration of the screening tools. The results show no significant differences among the cadres who improved when administering the screening tools. Fourteen (41%) primary healthcare workers (one general medical doctor, ten nurses, and three adherence counsellors) demonstrated no change, and H-NCI screening skills declined among eight (24%) primary healthcare workers (one general medical doctor, two nurses, and five adherence counsellors).

Eight (24%) healthcare workers (one general medical doctor, six nurses, and one adherence counsellor) showed improvements on the CAT-rapid, while five (15%) healthcare workers (one general medical doctor, two nurses, and two adherence counsellors) scored lower than they did immediately after the training. Fifty-nine percent (*n* = 20) of health care workers (14 nurses and six adherence counsellors) showed no change. The IHDS was demonstrated with no changes by thirteen (38%) healthcare workers (ten nurses and three adherence counsellors). Thirteen (38%) healthcare workers (one general medical doctor, nine nurses, and three adherence counsellors) improved when demonstrating the IHDS. There were eight (24%) healthcare workers who scored lower than they did immediately after the training (one general medical doctor, three nurses, and four adherence counsellors). There were no statistically significant differences among the three cadres.

### 3.4. Healthcare Workers' Confidence Using a Screening Tool

Healthcare workers who had limited or no knowledge of H-NCI symptoms or screening tools prior to the training were able to describe the symptoms of H-NCI (*M* = −1.27, *SD* = 1.14), identify H-NCI symptoms (*M* = −1.35, *SD* = 1.26), and felt more confident using an H-NCI screening tool after attending the Primary Healthcare H-NCI training (*M* = −1.59, *SD* = 1.05). This was measured after the second session of the training.

## 4. Discussion

To our knowledge, this study is the first to assess the viability of H-NCI screening by general medical doctors, nurses, and adherence counsellors at a primary healthcare level, in low- and middle-income countries. This study also focuses on the acceptability of in-field H-NCI training in a clinic setting. There were significant improvements in H-NCI knowledge among all three cadres following the training intervention, and this was sustained by most healthcare workers at eight weeks. Although only a few general medical doctors and nurses were able to administer the H-NCI screening tools with 100% accuracy immediately after training, several others made minor errors which improved over time. Junior nurses and adherence counsellors demonstrated greater difficulty with the administration of the screening tool, especially at eight weeks. Primary healthcare workers were in favour of attending future training that was brief and in-field, like the design adopted by the Primary Healthcare H-NCI training.

H-NCI knowledge improved among all three cadres of primary healthcare workers following the training intervention [10]. These preliminary findings are important for two reasons. First, there are extreme shortages of trained professionals in low-income and middle-income countries and second, there is a paucity of literature describing the adoption of task-sharing for H-NCI screening [28, 29]. Our training intervention was helpful as it provided clarity that H-NCI had not been addressed during previous primary healthcare worker HIV training. This study highlights that once equipped with knowledge of H-NCI, general medical doctors, nurses, and adherence counsellors could potentially fill in the skills gap by task-sharing the identification of neurocognitive or functional challenges among PWH. Each of these cadres would also be able to flag patients for further investigation at the facility or for referral to the next level of care among those with severe H-NCI. We hypothesize that the inclusion of H-NCI in future training, particularly HIV training targeting primary healthcare workers, may serve as a potential mechanism for bridging the neurocognitive skills gap in low-income and middle-income countries.

The viability of H-NCI training by nonspecialist healthcare workers at a primary healthcare level is still unclear. Our findings show that the administration of the H-NCI screening tools varied between general medical doctors, nurses, and adherence counsellors, similar to other local research comparing adherence counsellors and nurses [30]. Few general medical doctors and senior or mid-level nurses were able to administer the screening tool correctly. Several others demonstrated the screening tool with minor errors. This was not unexpected given that H-NCI screening tools differ from general clinical procedures or checklist-type examinations. H-NCI screening tools are interactive and require healthcare workers to master and perform demonstrations, as well as time tasks performed by patients. We saw several improvements in the administration of H-NCI screening tools among this group over time, suggesting that with additional time and mentorship, nonspecialist healthcare workers may be able to accurately administer H-NCI screening tools.

Several other junior nurses, including staff nurses or certified nursing assistants, as well as adherence counsellors experienced more challenges with the H-NCI screening tools. This did not improve over time. These cadres were only able to administer subsections of the screening tools, despite multiple practice sessions. Although staff nurses, certified nursing assistants, and adherence counsellors may have more contact with patients, certain diagnostic and treatment activities are conducted by midlevel nurses such as registered nurses or general medical doctors. Thus, these cadres may not be accustomed to tools that follow stringent processes such as those required in H-NCI screening tools.

Among this group, nurses and adherence counsellors struggled with demonstrating motor-function activities, including the finger tapping test and the alternating hand sequence test. These activities require healthcare workers to master the finger tap and hand sequences themselves, before demonstrating this to a patient. Primary healthcare workers also struggled with tasks that required simultaneous or multiple actions, such as the use of the timer or counting correct sequences. Inaccuracy in administering H-NCI screening tools may lead to high false positive and negative rates. This may in turn result in increased anxiety and poor quality of life among PWH. Since H-NCI screening tools require precision to be accurate, junior or lay healthcare workers may not be appropriate. Tools that do not rely heavily on motor-function demonstrations, such as the HIV Cognitive Symptom Questionnaire, which involves a series of questions, may be more suitable for these cadres [31]. Alternatively, these healthcare worker cadres could flag at risk patients using checklists or question-based screening tools for further investigation by general medical doctors or senior nurses who may be more appropriate to administer an H-NCI screening tool.

The appropriateness of the H-NCI training targeting primary healthcare workers was mixed. The training received a positive response from primary healthcare workers, and knowledge improved among all cadres. Healthcare workers found the training design appropriate and acceptable for use in busy clinic settings. The training was designed to reduce the impact on healthcare services by preventing healthcare workers from leaving the facility for several hours. The use of the weekly staff meeting time slot also allows for future on-going training sessions to be implemented feasibly. The on-site training design was feasible, especially in overburdened clinics with limited staff, as healthcare workers were close enough to address emergency queries. However, this is also a limitation to the design as the training session was subject to disruptions, as healthcare workers were requested to provide patient care while the training was underway. Due to the complexity of the H-NCI screening tools, the skill component of the training may require more time and mentoring to ensure that screening is mastered. Although screening may be practical, questions regarding the clinical utility and the timing of such screening remain. Further investigation into training methods for midlevel or lay healthcare workers in the administration of H-NCI screening tools is also required.

## 5. Limitations

This study has several limitations. The pilot sample was confined to one region, in a single district of KwaZulu-Natal, and may not be generalized to other parts of the country or other countries with limited resources. Due to the small sample size, our findings are not generalizable. Our statistical analyses were limited to descriptive statistics. Despite efforts to create a suitable in-facility learning environment, there were several challenges including postponements of the training due to healthcare workers being required to administer the COVID-19 vaccines, interruptions for urgent patient care, as well as logistical or infrastructural challenges which may have indirectly impacted learning. A final limitation of this pilot study was the single eight-week follow-up period. Repeated follow-up assessments would provide a better indication of the uptake of knowledge and whether skills were sustained over time.

## 6. Conclusion

H-NCI knowledge improved among primary healthcare workers and was retained after eight weeks. Skills training, however, presented differences and challenges. On-going training could be delivered in person or using hybrid platforms for various cadres of primary healthcare workers. Skill training, where direct observation, correction, and supervision are required, will need further research. Future research could explore whether alternate screening tools may be more suitable for general medical doctors and senior nurses to administer in busy primary healthcare clinics [31–36]

## Figures and Tables

**Table 1:** Accuracy of healthcare workers' HIV-associated neurocognitive impairment screening tool delivery.

Screening tool	Task% (score)	Doctor (*n* = 2)	Nurse (*n* = 22)	Adherence counsellor (*n* = 10)	Total (*n* = 34)	Std. deviation	Minimum	Maximum	Chi-square	Df	-value
IHDS	Motor speed	88 (14)	83 (13)	64 (10)	77 (12)	4,2	3,0	16,0	35,4	22,0	0.07
	Psychomotor speed	89 (16)	77 (14)	66 (12)	75 (13)	4,6	4,0	18,0	20.65	20	0.41
	Memory recall	92 (11)	77 (9)	62 (7)	73 (9)	3,1	2,0	12,0	39.29	16	0.002
	IHDS total	90 (43)	82 (39)	57 (28)	75 (13)	11,4	11,0	48,0	36.47	32	0.31

CAT-R	Symptoms	100 (6)	94 (5)	100 (6)	96 (6)	0,6	3,0	6,0	3.97	4	0.33
	Trail making	88 (7)	76 (6)	56 (5)	71 (6)	2,2	1,0	8,0	10.77	14	0.72
	Motor speed	91 (15)	79 (13)	67 (11)	76 (12)	4,6	2,0	16,0	24.93	22	0.34
	Psychomotor speed test	81 (15)	70 (13)	61 (11)	68 (12)	5,4	3,0	18,0	12.42	14	0.55
	Memory recall	95 (10)	87 (9)	64 (6)	81 (8)	2,4	1,0	10,0	41.79	36	0.28
	CAT-rapid total	90 (58)	84 (54)	66 (42)	79 (51)	13,7	18,0	64,0	34.62	40	0.76

Total combined score		91 (101)	85 (93)	64 (70)	78 (86)	24,0	29,0	110,0	38.02	42	0.73

**Table 2:** Accuracy of healthcare workers' HIV-associated neurocognitive impairment screening tool delivery two months posttraining.

Cadre		Doctor (*n* = 2)			Nurse (*n* = 22)			Adherence counsellor (*n* = 10)			Total (*n* = 34)			Chi-square	Df	*p*-value
Screening tool	Task	No change	Improved	Declined	No change	Improved	Declined	No change	Improved	Declined	No change	Improved	Declined			
IHDS	Motor speed	5.9 (2)	0 (0)	0 (0)	55.9 (19)	5.9 (2)	2.9 (1)	17.6 (6)	0 (0)	12 (4)	79.4 (27)	5.9 (2)	14.7.8 (5)	7.99	4	0.09
	Psychomotor speed	0 (0)	2.9 (1)	2.9 (1)	35.3 (12)	23.5 (8)	5.9 (2)	17.6 (6)	5.9 (2)	5.9 (2)	52.9 (18)	32.4 (11)	5.9 (5)	4,29	4	0.37
	Memory recall	5.9 (2)	0 (0)	0 (0)	35.3 (12)	23.5 (8)	5.9 (2)	14.7 (5)	5.9 (2)	8.8 (3)	55.9 (19)	29.4 (10)	8.8 (5)	4,37	4	0.36
	IHDS total	0 (0)	2.9 (1)	2.9 (1)	29.4 (10)	26.5 (9)	8.8 (3)	8.8 (3)	8.8 (3)	11.8 (4)	38.2 (13)	38.2 (13)	23.5 (8)	4.20	4	0.38

CAT-rapid	Symptoms	5.9 (2)	0 (0)	0 (0)	41.2 (14)	20.6 (7)	2.9 (1)	11.8 (4)	8.8 (3)	8.8 (3)	55.8 (20)	29.4 (10)	11.8 (4)	5.6	4	0.20
	Trail making	5.9 (2)	0 (0)	0 (0)	41.2 (14)	17.6 (6)	5.9 (2)	20.6 (7)	2.9 (1)	5.9 (2)	67.6 (23)	20.6 (7)	11.8 (4)	2.75	4	0.60
	Motor speed	2.9 (1)	0 (0)	2.9 (1)	26.5 (9)	32.4 (11)	5.9 (2)	17.6 (6)	5.9 (2)	5.9 (2)	47.1 (16)	38.2 (13)	14.7 (5)	5.32	4	0.26
	Psychomotor speed test	0 (0)	2.9 (1)	2.9 (1)	41.2 (14)	17.6 (6)	5.9 (2)	17.6 (6)	2.9 (1)	8.8 (3)	58.8 (20)	23.5 (8)	17.6 (6)	4.46	4	0.35
	Memory recall	0 (0)	2.9 (1)	2.9 (1)	41.2 (14)	17.6 (6)	5.9 (2)	17.6 (6)	2.9 (1)	5.9 (2)	58.5 (20)	23.5 (8)	17.6 (6)	4.20	4	0.38
	CAT-rapid total	0 (0)	2.9 (1)	2.9 (1)	41.2 (14)	17.6 (6)	5.9 (2)	17.6 (6)	2.9 (1)	5.9 (2)	58.5 (20)	23.5 (8)	17.6 (6)	4.20	4	0.38

Total combined score	Total	0 (0)	2.9 (1)	2.9 (1)	29.4 (10)	29.4 (10)	5.9 (2)	5.9 (2)	8.8 (3)	14.7 (5)	35.3 (12)	41.2 (14)	23.5 (8)	7.97	4	0.09

## Data Availability

Due to privacy and ethical concerns, neither the data nor the source of the data can be made available.

## Additional Points

Summary. What is already known about this topic? (i) HIV-associated neurocognitive impairment (H-NCI) remains a common comorbidity; however, there are shortages of appropriately skilled healthcare workers able to identify and manage this disorder in low-income and middle-income countries. (ii) People living with HIV receive the majority of their care at a primary healthcare level. (iii) Screening for H-NCI at a primary healthcare level in South Africa is uncommon, and the reasons for this remain unclear. This is despite most people living with HIV accessing HIV services at this level of care. What this paper adds (research findings and key new information). (i) This paper provides preliminary evidence needed to explore task-sharing of H-NCI screening in low-income and middle-income countries. (ii) This paper demonstrates that with training, general medical doctors and senior nurses can administer H-NCI screening tools. The implications of this paper are (how findings influence or can be used to change policy/practice/research/education). (i) These findings provide preliminary evidence needed to inform H-NCI training interventions targeting primary healthcare workers, including general medical doctors and senior nurses.

## Supplementary Materials

Supplementary Materials Supplementary Table S1: summary of core knowledge aptitudes and specific focus skills. Supplementary Table S2: HIV-associated NCI training knowledge, attitudes, and views pre-test and post-test training questionnaire. Please tick the response that best describes your views in the following table.
