# Supplementary material for: Integrating HIV-Associated Neurocognitive Impairment Screening within Primary Healthcare Facilities: A Pilot Training Intervention
Source: Nurs Res Pract. 2022 Aug 13;2022:4495586. doi: 10.1155/2022/4495586 (PMC9392617; doi:10.1155/2022/4495586)
Supplement: Supplementary Materials — Supplementary Table S1: summary of core knowledge aptitudes and specific focus skills. Supplementary Table S2: HIV-associated NCI training knowledge, attitudes, and views pre-test and post-test training questionnaire. Please tick the response that best describes your views in the following table. [file 4495586.f1.docx]

**Supplementary Material**

**Supplementary Table S1**

*Summary of core knowledge aptitudes and specific focus skills*

| Existing knowledge |
| --- |
| The Human Immunodeficiency Virus (HIV) |
| HIV and the central nervous system |
| Core knowledge aptitudes |
| HIV-associated NCI |
| A brief history of HIV-associated NCI |
| Risk factors to be considered when investigating HIV-associated NCI |
| Characteristics of HIV-associated NCI |
| Treatment non-adherence and HIV-associated NCI |
| Co-morbidities and HIV-associated NCI |
| Difficulties in using these criteria at the primary healthcare level |
| Managing patients with symptomatic HIV-associated NCI |
| Consequences of HIV-associated NCI |
| Specific Skills |
| Administering and interpreting the International HIV-Dementia Scale |
| Administering and interpreting the CAT-Rapid |

**Supplementary Table S2**

*HIV-associated NCI training knowledge, attitudes and views pre and post-test training questionnaire*

| Question | | A | B | C | D |
| --- | --- | --- | --- | --- | --- |
|  | HIV is able to impact the brain | True | False |  |  |
|  | Which of the following is not part of the HIV-associated CI spectrum | HIV-associated dementia | Mild neurocognitive disorder | Bi-polar disorder | All of the above |
|  | HIV-associated CI is no longer a problem among PWH | True | False |  |  |
|  | A PWH with HIV-associated CI may complain of difficulties with | Suicidal thoughts | Hearing voices, seeing things or feeling angry | Memory, learning, attention, problem solving planning, decision making | None of the above |
|  | Substance abuse and alcohol abuse are caused by cognitive impairment | True | False |  |  |
|  | My patient who is ART naïve and has a low CD4 count will not be at risk for CI | True | False |  |  |
|  | Which of the following tools can be used to detect CI among PWH | IHDS | Depression scale | Screening Tool for Mental Health Disorders | Self-report |
|  | Mild CI can get worse if my patient is non-adherent to ART | True | False |  |  |
|  | Which of the following are examples of a HIV-associated CI screening tool | International HIV-dementia scale | Becks Depression scale | CT scan | Lumbar puncture |
|  | HIV-associated CI screening tools measure cognitive functions | True | False |  |  |

Please tick the response that best describes your views in the table below.

| Question | | Strongly disagree | Disagree | I do not know enough about this to answer | Agree | Strongly agree |
| --- | --- | --- | --- | --- | --- | --- |
|  | I am able to explain how HIV affects the brain |  |  |  |  |  |
|  | I am able to describe the symptoms of cognitive impairment among people living with HIV |  |  |  |  |  |
|  | I learned about cognitive impairment among people living with HIV in my previous training |  |  |  |  |  |
|  | I have heard previously that HIV can affect the brain |  |  |  |  |  |
|  | I am able to identify cognitive impairment among people living with HIV |  |  |  |  |  |
|  | I believe that it is important to know about cognitive impairment among PWH |  |  |  |  |  |
|  | I feel confident using a cognitive impairment screening tool among my patients |  |  |  |  |  |
|  | I believe that a cognitive impairment screening tool will benefit my patients |  |  |  |  |  |
|  | I would like to have access to a cognitive impairment screening tool at my clinic |  |  |  |  |  |
|  | Screening for cognitive impairment among people living with HIV is unnecessary |  |  |  |  |  |
